# Expression of high mobility group B1 and toll-like receptor-nuclear factor κB signaling pathway in chronic subdural hematomas

**DOI:** 10.1371/journal.pone.0233643

**Published:** 2020-06-01

**Authors:** Koji Osuka, Yasuo Watanabe, Nobuteru Usuda, Kenichiro Iwami, Shigeru Miyachi, Masakazu Takayasu

**Affiliations:** 1 Department of Neurological Surgery, Aichi Medical University Nagakute, Aichi, Japan; 2 High Technology Research Center, Pharmacology, Showa Pharmaceutical University, Machida, Tokyo, Japan; 3 Department of Anatomy II, Fujita Health University School of Medicine, Toyoake, Aichi, Japan; MHH (Hannover Medical School), GERMANY

## Abstract

Chronic subdural hematoma (CSDH) is an angiogenic and inflammatory disease. Toll-like receptors (TLRs) transduce intracellular signals, resulting in the activation of nuclear factor κB (NF-κB), which leads to the production of inflammatory cytokines. High-mobility group box 1 (HMGB1) functions as a mediator of inflammatory responses through TLRs. In this study, we examined the expression of HMGB1 and components of the Toll-like receptor and NF-κB signaling pathways in the outer membrane of CSDH. Eight patients whose outer membrane was successfully obtained during trepanation surgery were included in this study. The expression of TLR4, myeloid differentiation factor 88 (MyD88), interleukin-1 receptor-associated kinase 4 (IRAK4), TNF receptor-associated factor 6 (TRAF6), TGFβ-activated kinase 1 (Tak1), interferon regulatory factors 3 (IRF3), IκB kinase β (IKKβ), IKKγ, IκBε, IκBα, NF-κB/p65 and β-actin was examined by Western blot analysis. The expression of TLR4, NF-κB/p65 and interleukin-6 (IL-6) was also examined by immunohistochemistry. The concentrations of HMGB1 and IL-6 in CSDH fluids were measured using ELISA kits. Above-mentioned molecules were detected in all cases. In addition, TLR4, NF-κB/p65 and IL-6 were localized in the endothelial cells of vessels within CSDH outer membranes. The concentrations of HMGB1 and IL-6 in CSDH fluids were significantly higher than that in the CSF and serum. There existed a correlation between the concentrations of HMGB1 and IL-6 in CSDH fluids. Our data suggest that HMGB1 in CSDH fluids produces the inflammatory cytokine IL-6 in endothelial cells through the Toll-like receptor and NF-κB signaling pathways. Anti-HMGB1 therapy might be a useful method to treat the growth of CSDH.

## Introduction

Angiogenesis and inflammation play an important role in the growth of chronic subdural hematoma (CSDH). A significantly higher concentration of the inflammatory cytokine interleukin-6 (IL-6) in CSDH fluids than that in serum has been reported [1, 2]. IL-6 induces the enlargement of the gap between endothelial cells, resulting in increased vascular permeability [3]. Moreover, the concentrations of the proinflammatory and inflammatory cytokines IL-6 and IL-8 are significantly increased in recurrent cases of CSDH [4, 5]. IL-6 activates the Janus kinase (JAK)/signal transducer and activator of transcription (STAT) signaling pathway in endothelial cells and fibroblasts in CSDH outer membranes [6, 7]. This signaling pathway may play important roles in the angiogenesis and growth of fibroblasts in the outer membrane. However, the mechanisms by which IL-6 is produced in CSDH fluids have not been fully clarified.

Members of the Toll-like receptor (TLR) family recognize conserved microbial structures, such as bacterial lipopolysaccharide (LPS), and are involved in both innate and adaptive immune responses to various microbial infections [8,9]. Recently, there has been increasing evidence supporting the role of TLRs in the pathogenesis of several neurodegenerative diseases. The TLR4-dependent upregulation of cytokines is involved in the progression of Alzheimer’s disease [10]. Microglia-mediated neuroinflammation may play an important role in the initiation and progression of dopaminergic (DA) neurodegeneration in Parkinson's disease (PD), and TLR4 induced by the prothrombin kringle-2 plays an essential role in the activation of microglia in the adult brain [11]. After subarachnoid hemorrhage, neuronal apoptosis and vasospasm depend upon the TLR4-myeloid differentiation factor 88 (MyD88) signaling pathway and microglia [12]. These data suggest that neuroinflammation through TLR4 plays an essential role in neurological disease. High-mobility group box 1 (HMGB1) functions as a mediator of inflammatory responses through TLRs.

The present study explored the mechanism by which IL-6 is produced in CSDH fluids with a focus on the HMGB1 and TLR signaling pathway. To explore this mechanism, we performed immunoblotting and immunohistochemical analyses of CSDH outer membranes.

## Materials and methods

### Patients

This study included eight patients (six men and two women; 59–90 years old; mean age, 78 years) with CSDH that was confirmed by computed tomography or magnetic resonance imaging. Burr hole irrigation and drainage surgery were performed on all patients under local anesthesia at Aichi Medical University Hospital. The Ethics Committee of Aichi Medical University approved this clinical experiment (2016-H137). Informed consent was obtained from each patient or patient’s family.

### Materials

All chemicals were obtained from Sigma Chemical (St. Louis, MO) unless otherwise specified.

### Western blotting analysis

The outer membranes of CSDHs were obtained during trepanation surgery and then homogenized in 80 μL of homogenization buffer containing 50 mM Tris base/HCl (pH 7.5), 0.1 mM dithiothreitol, 0.2 mM EDTA, 0.2 mM EGTA, 0.2 mM phenylmethylsulfonyl fluoride, 1.25 μg/mL pepstatin A, 0.2 μg/mL aprotinin, 1 mM sodium orthovanadate, 50 mM sodium fluoride, 2 mM sodium pyrophosphate, and 1% Nonidet P-40. The homogenates were centrifuged at 12,000 × g for 10 min at 4°C. The protein concentrations in the supernatants were determined using a Bradford assay with bovine serum albumin as the standard. The crude samples (25 μg of protein in each sample) were separated using 7.5% sodium dodecyl sulfate (SDS)-polyacrylamide gel electrophoresis. The proteins were transferred to polyvinylidene difluoride membranes and incubated with primary antibodies against β-actin (#A2066, Sigma), TLR4 (#sc-293072, Santa Cruz Biotechnology, Dallas, TX), MyD88 (#sc-74532, Santa Cruz Biotechnology), interleukin-1 receptor-associated kinase 4 (IRAK4; #4363, Cell Signaling Technology, Danvers, MA), TNF receptor-associated factor 6 (TRAF6; #8028, Cell Signaling Technology), TGFβ-activated kinase 1 (Tak1; #sc-166562, Santa Cruz Biotechnology), interferon regulatory factors 3 (IRF3; #4962, Cell Signaling Technology), IκB kinase β (IKKβ; #187320, Transduction Laboratory, San Jose, CA), IKKγ (#189920, Transduction Laboratory), IκBε (#195020, Transduction Laboratory), IκBα (#158220, Transduction Laboratory) and nuclear factor κB/p65 (NF-κB/p65; #8242, Cell Signaling Technology), with each diluted 1:750, overnight at 4°C. After being washed, the membranes were incubated with secondary antibodies conjugated to horseradish peroxidase (Sigma) diluted 1:3000 for 30 min at room temperature. The reactions were developed with ECL Plus (GE Healthcare, Buckinghamshire, UK). Untreated Jurkat cells were lysed in CHAPS cell extract buffer, and the cytoplasmic fraction was used as the positive control.

### Histological examinations

To analyse the cellular localization of TLR4, NF-κB/p65 and IL-6, immunohistochemical staining was performed on samples from three patients at room temperature using the avidin-biotinylated peroxidase complex (ABC) technique. To preserve the outer membranes of the CSDH samples, the samples were incubated in 10 mL of ice-cold 4% paraformaldehyde in 0.1 M phosphate buffer (pH 7.4) for 3 h. Serial axial cryostat sections (10 μm) were placed on slides for staining. Non-specific immunoreactivity was blocked by incubation with goat serum for 30 min. The samples were treated with primary antibodies against TLR4 (#sc-293072, Santa Cruz Biotechnology) diluted 1:20, NF-κB/p65 (#8242, Cell Signaling Technology) diluted 1:500 and interleukin-6 (IL-6; #12153, Cell Signaling Technology) diluted 1:50 overnight at 4°C. After being washed, the samples were incubated with biotinylated anti-mouse or rabbit IgG for 1 h and then ABC for 1 h. Sera used for the blocking step, biotinylated antibodies, and ABC were purchased from Vector Laboratories (Burlingame, CA). The reaction products were developed by incubating the sections in 0.05% 3,3'-diaminobenzidine tetrachloride and 0.01% H_2_O_2_ in 50 mM Tris-HCl (pH 7.5) for 10 min. The histopathological analyses without these antibodies were used as negative controls.

### Analysis of HMGB1 and IL-6

CSDH fluids from 20 patients were sampled during trepanation surgery including the eight patients whose outer membranes were studied. There were 14 men and 6 women in this analysis, ranging in age from 50–92 years, with an average age of 80 years. As a control, CSF samples were obtained from 7 patients undergoing neck clipping for unruptured cerebral aneurysm. Serum samples were collected from 6 healthy voluntary adults. After collection, all samples were immediately centrifuged, and the supernatant fluids were stored at -80˚C until analysis. HMGB1 and IL-6 levels were measured using an enzyme-linked immunosorbent assay (ELISA; Abbexa, Cambridge, UK and R&D Systems, Inc., Minneapolis, MN, respectively) according to the manufacturer’s instructions. The limits of detection for HMGB1 and IL-6 in these assays were 0.1 ng/ml and 0.7 pg/ml, respectively.

### Statistical analysis

Data are expressed as the mean ± SE. Significant differences between the groups were assessed using one-way analysis of variance (ANOVA), followed by Bonferroni/Dunn test for multiple comparisons. Significance was indicated when p < 0.05.

## Results

### Clinical data

Clinical data are presented in Table 1. Seven patients had a history of mild head injury without any hemostatic disorder, and in the remaining patient, there was no episode of apparent head trauma. Two patients had received antiplatelets for myocardial infarction or anticoagulation therapy for atrial fibrillation. Some patients had hypertension, but none had been prescribed β-blockers or angiotensin-converting enzyme inhibitors. The Glasgow Outcome Scale showed good recovery to previous levels of daily activity for all patients, except for one with dementia.

**Table 1:** Clinical data of 8 patients with chronic subdural hematoma.

Case	Age, Gender	mechanism of injury	Trauma (weeks ago)	GCS	Symptoms	comorbidity	medication	GOS
1	77, M	fall down	8	13	hemiparesis	DM, HT, MI	sitagliptin, amlodipine, aspirin	GR
2	77, M	fall down	6	15	headache	gastric cancer	none	GR
3	59, M	fall down	7	15	headache	none	none	GR
4	90, M	fall down	8	14	hemiparesis	dementia, HT, HL	amlodipine, pitavastatin, donepezil	MD
5	85, M	fall down	10	15	headache	atrial fibrillation	warfarin	GR
6	75, M	traffic accident	8	14	gait disturbance	none	none	GR
7	80, F	unknown	-	15	epilepsy	HT, HL	atorvastatin, amlodipine	GR
8	83, F	fall down	7	14	hemiparesis	HT	azilsartan	GR

GCS, Glasgow Coma Scale at admission; GOS, Glasgow Outcome Scale 3 months after operation

GR, good recovery; MD, moderate disability; M, male; F, female

DM, diabetes mellitus; HL, hyperlipidemia; HT, hypertension

### Western blotting analysis of TLR4 and NF-κB signaling pathway

**Fig 1:**
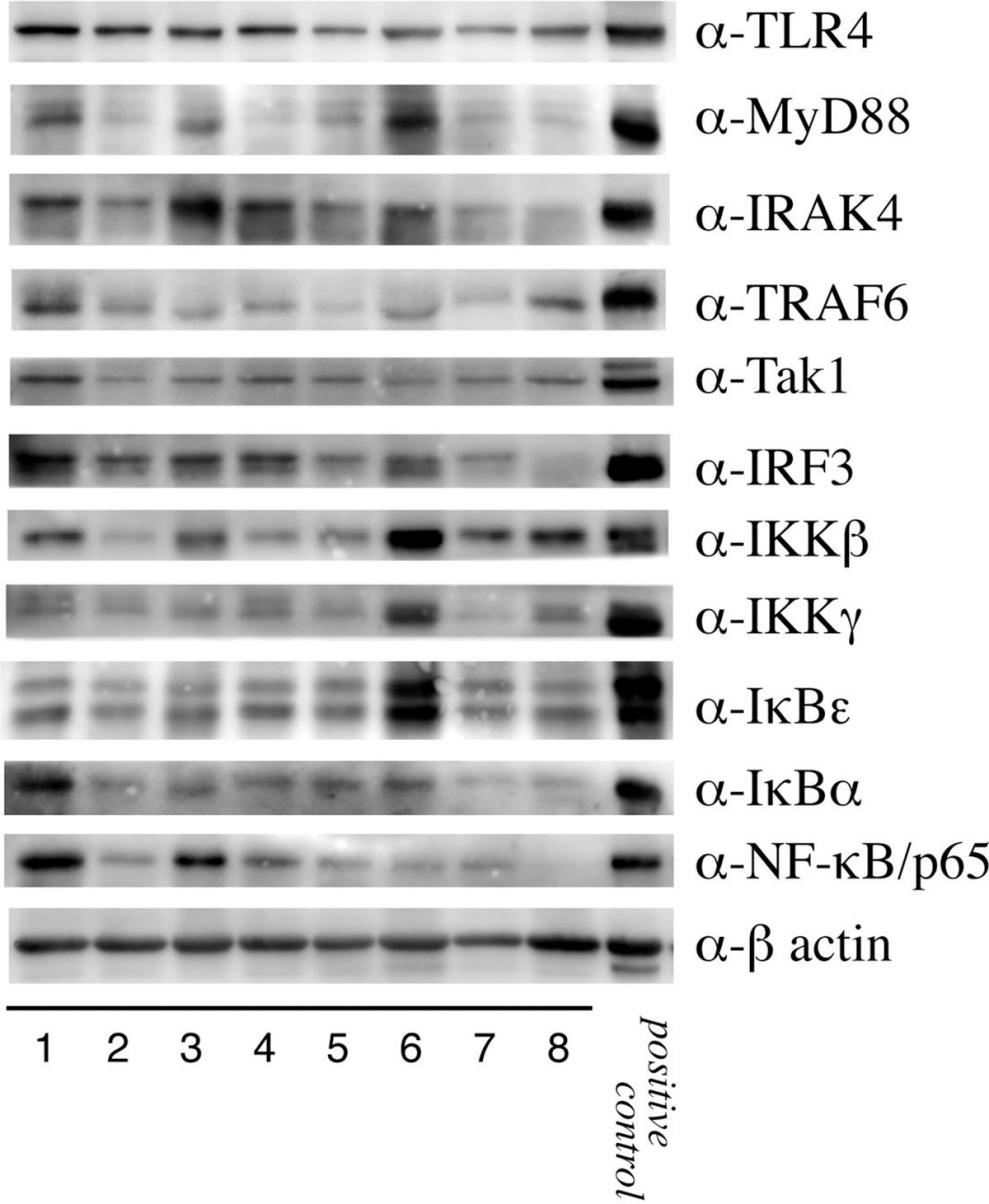
Western blotting showing the expression of proteins in the outer membranes of chronic subdural hematomas. The membranes were homogenized in homogenization buffer, and the supernatants were subjected to Western blotting with anti-Toll-like receptor 4 (α-TLR4), anti-myeloid differentiation factor 88 (α-MyD88), anti-interleukin-1 receptor-associated kinase 4 (α-IRAK4), anti-TNF receptor-associated factor 6 (α-TRAF6), anti-TGFβ-activated kinase 1 (α-Tak1), anti-interferon regulatory factor 3 (α-IRF3), anti-IκB kinase β (α-IKKβ), anti-IκB kinase γ (α-IKKγ), anti- IκBε (α-IκBε), anti-IκBα (α-IκBα), anti-nuclear factor κB/p65 (α- NF-κB/p65) and anti-β-actin (α-β-actin) antibodies. Notably, all molecules involved in the TLR and NF-κB pathways were detected in almost all cases. Positive control: positive control using Jurkat cells.

The levels of detected β-actin were homogeneous in all samples, which suggested that equal amounts of protein were applied to SDS gels. TLR4, MyD88, IRAK4, TRAF6, Tak1, IRF3, IKKβ, IKKγ IκBε, IκBα and NF-κB/p65 were detected in nearly all cases, although in some cases, their signals were weak (Fig 1). However, Western blotting analysis using positive controls revealed that these molecules were correctly detected (Fig 1).

### Histological observations

**Fig 2:**
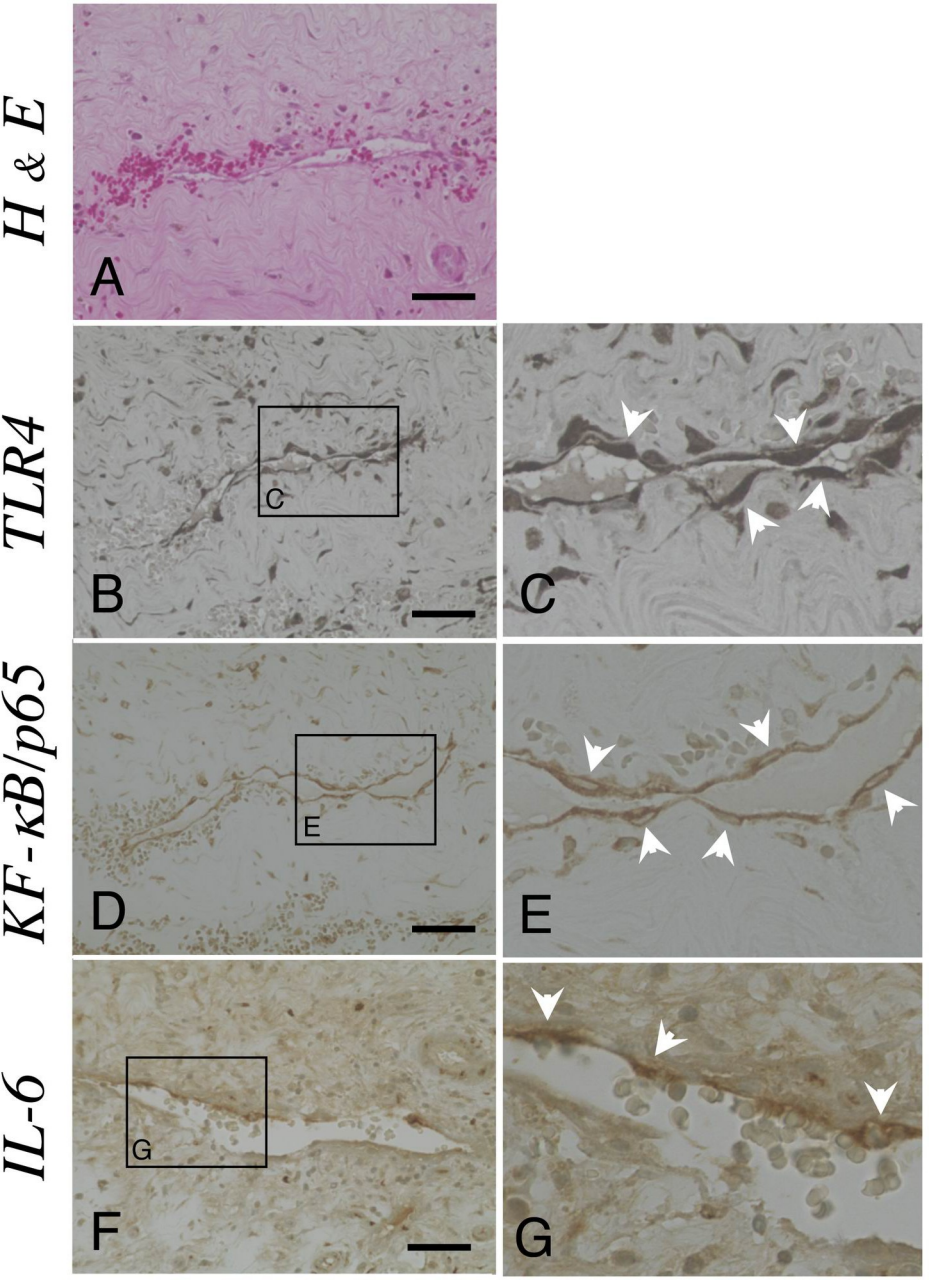
The expressions of TLR4, NF-κB/p65 and IL-6 within the outer membranes. (A) The presence of fibroblasts, collagenous fibers, inflammatory cells and vessels was confirmed by hematoxylin and eosin (H & E) staining. Ten-micrometer slices were immunostained with polyclonal antibodies against Toll-like receptor 4 (TRL4, B and C), nuclear factor-κB/p65 (NF-κB/p65, D and E) and interleukin-6 (IL-6, F and G) using the ABC method. The areas within the rectangle, labeled B, D and F, are shown at a higher magnification in panels C, E and G, respectively. Note that these molecules were expressed in endothelial cells (arrowheads). Scale bars = 50 μm.

Hematoxylin and eosin staining of CSDH membranes showed well-developed vessels and fibroblasts between collagenous fibers (Fig 2A). TLR4 (Fig 2B and 2C), NF-κB/p65 (Fig 2D and 2E) and IL-6 (Fig 2F and 2G) were localized in the endothelial cells of vessels within the outer membranes. Note that these molecules are expressed in the endothelium (Fig 2C, 2E and 2G, arrowheads). In the negative controls examined without primary antibodies, the endothelial cells were consistently negative for the markers listed above.

### Concentrations of HMGB1 and IL-6

**Fig 3:**
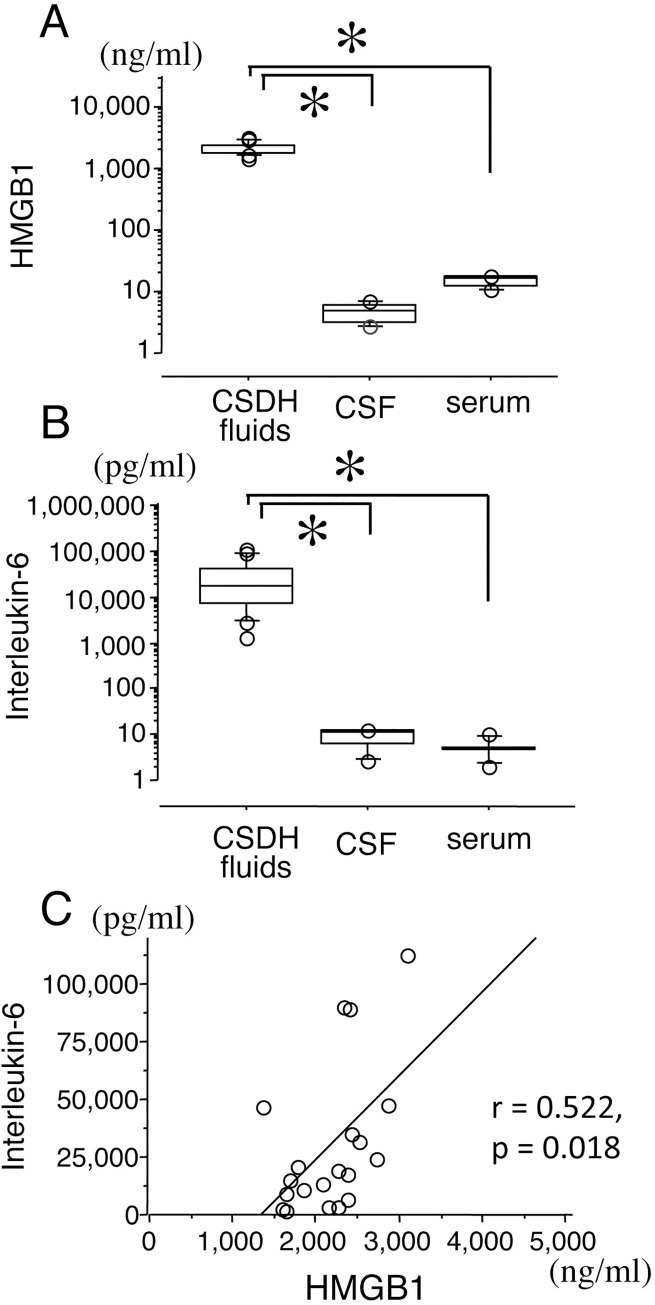
Concentrations of HMGB1 and IL-6 protein. Concentrations of high-mobility group B1 **(**HMGB1) and interleukin-6 **(**IL-6) in chronic subdural hematoma (CSDH, n = 20), control cerebrospinal fluid (CSF, n = 7) and control serum (n = 6) (A and B, respectively). Concentrations were measured with an ELISA kit. Data represent the median values, 25^th^ and 75^th^ percentiles with maximum/minimum whiskers. The correlation between the concentrations of HMGB1 and IL-6 according to Pearson's correlation coefficient (C). *p < 0.05 by one-way ANOVA, followed by Bonferroni/Dunn test.

In the control CSF, serum samples and CSDH fluids, the mean HMGB1 concentrations were 4.7 ± 0.6 ng/mL, 15.0 ± 1.3 ng/mL and 2176.9 ± 103.9 ng/mL, respectively (Fig 3A). In the control CSF, serum samples and CSDH fluids, the mean IL-6 concentrations were 9.2 ± 1.5 pg/mL, 5.3 ± 1.1 pg/mL and 29,961.8 ± 7,203.4 pg/mL, respectively (Fig 3B). The mean concentrations of HMGB1 and IL-6 in CSDH fluids were significantly higher than those in control CSF and serum (Fig 3A and 3B). The concentration of HMGB1 in CSDH fluids was positively correlated with that of IL-6, according to Pearson’s correlation coefficient (r = 0.522, p = 0.018, Fig 3C).

## Discussion

In this study, we identified the expression of TLR and NF-κB signaling molecules in CSDH outer membranes by Western blot analysis for the first time. Immunohistochemical analysis showed that TLR4, NF-κB/p65 and IL-6 were primarily expressed in endothelial cells. Moreover, the concentration of HMGB1 was significantly increased in CSDH fluids compared to that in the CSF and serum.

TLR is known to signal through the NF-κB pathway as well as mitogen-activated protein kinases (MAPKs) and subsequently regulate immune and inflammatory genes [13]. MyD88 is a cytoplasmic adaptor protein of TLR. Endometrial cells play an intrinsic role in the innate immune system via TLR4/MyD88-dependent pathways [14]. The association between TLR4 and MyD88 recruits and activates IRAK4 [15], which forms a complex with TRAF6, activating Tak1 and IKK and resulting in the eventual activation of the NF-κB and MAPK pathways [16, 17]. The activation of TLR4 induces the expression of NF-κB-controlled genes encoding the inflammatory cytokines IL-1, IL-6 and IL-8 (9). Cerebral ischemia/reperfusion activates the TLR4-mediated MyD88-dependent pathway and upregulates NF-κB [18]. Electroacupuncture alleviates cerebral inflammation in cerebral ischemia/reperfusion injury through the TLR4/NF-κB pathway, which is accompanied with the suppressed secretion of the inflammatory cytokines TNF-α, IL-1β and IL-6 [19]. Two major LPS pathways have been suggested: MyD88-dependent and MyD88-independent pathways. The expression of IFN-β induced by TLR4 occurs through a MyD88-independent pathway that activates IRF-3, a key transcription factor for the induction of IFN genes [20]. The expression of these major TLR signaling pathway molecules was confirmed by Western blot analysis and immunohistochemistry, suggesting that the TLR signaling pathway might play an important role in the growth of the CSDH outer membrane through the NF-κB signaling pathway.

Previous studies have shown that wall shear stress and bacterial lipopolysaccharides induce the release of interleukin-6 by endothelial cells (ECs) [21, 22]. Continuous mechanical stretching induces IL-6 secretion from ECs following the sequential activation of IKKs and NF-κB [23]. The IKK/IκB/NF-κB signaling pathway is essential and sufficient for pro-inflammatory gene expression in primary endothelial cells [24]. The promoter region of the IL-6 gene has a putative NF-κB-binding site. Therefore, NF-κB is involved in the control of the IL-6 gene, which is activated in chronic inflammatory diseases [25, 26]. LPS activates human intrahepatic biliary epithelial cells through the TLR4-NF-κB and TLR4-MAPK signaling pathways in inflammatory biliary cirrhosis, resulting in the production of IL-6 and IL-8 [27]. That IKKβ, IKKγ, IkBα, IkBε and NF-κB/p65 were detected by Western blot analysis and NF-κB/p65 and IL-6 were detected in vessel endothelial cells by immunohistochemistry suggests that the IKK/IκB/NF-κB signaling pathway also plays an important role in the production of inflammatory cytokines in CSDH fluids.

**Fig 4:**
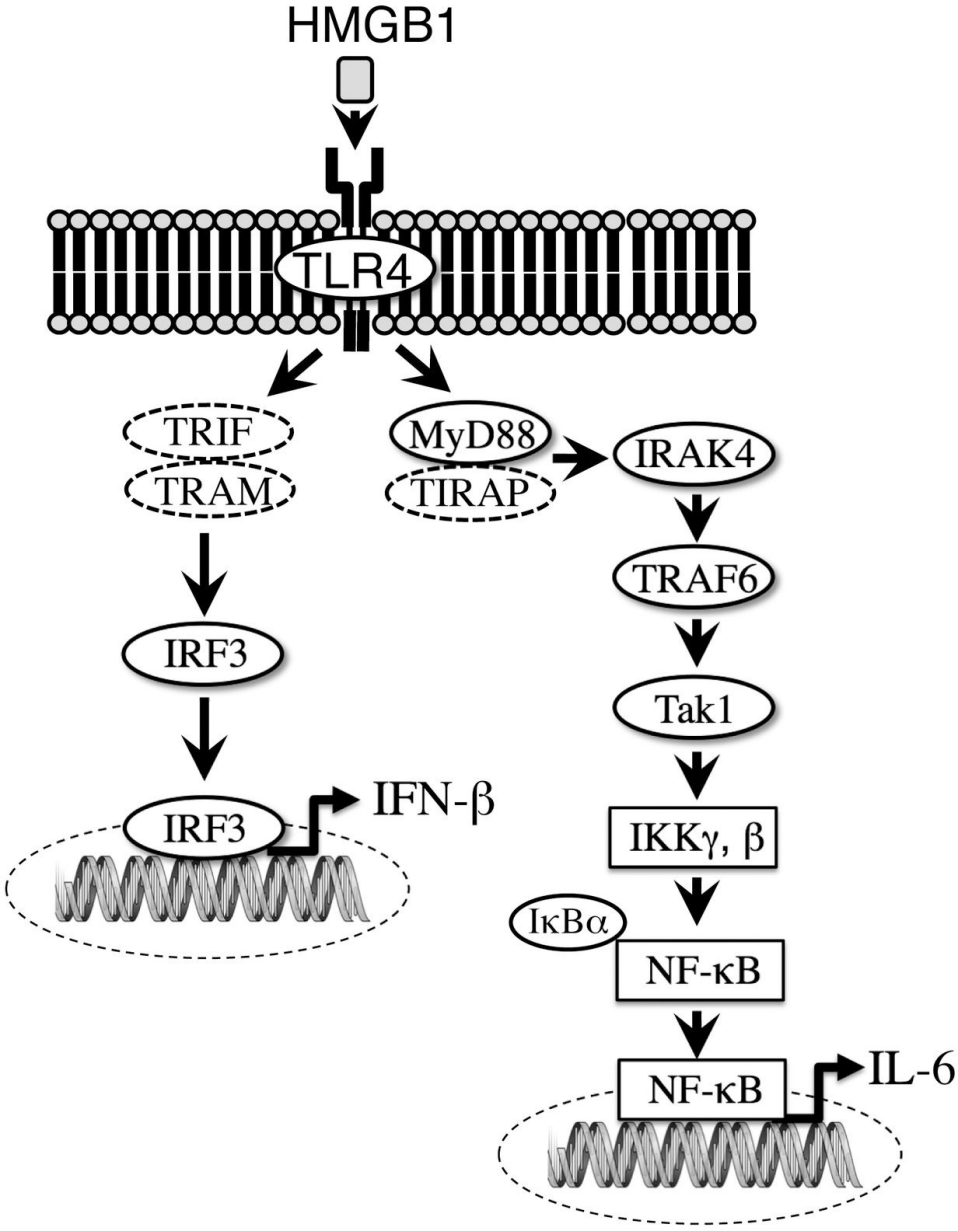
Mechanism of TLR4 signal transduction in the outer membrane of chronic subdural hematoma. High concentrations of high-mobility group box 1 (HMGB1) protein in the chronic subdural hematoma (CSDH) fluids activate Toll-like receptor 4 **(**TLR4) in the endothelial cells within the CSDH outer membranes, producing interleukin-6 (IL-6) through the TLR/NF-κB pathway. TRIF; Toll-receptor-associated activator of interferon, TRAM; Toll-receptor-associated molecule, TIRAP; Toll/interleukin-1 receptor-associated protein.

HMGB1 was originally identified as an architectural DNA-binding protein and a ubiquitous, abundant nuclear protein [28]. HMGB1 is released by necrotic or damaged cells but not apoptotic cells and is a potent mediator of inflammation [29]. HMGB1 induced cellular activation and NF-κB-dependent transcription through TLR2 and TLR4 [30–32]. Furthermore, HMGB1 has been shown to play important roles in the induction of inflammatory diseases. HMGB1 exaggerated brain infarction through the activation of inflammatory responses in the ischemic region [33]. A remarkable reduction in cerebral infarction was accomplished upon treatment with neutralizing anti-HMGB1 monoclonal antibody (mAb) [33]. Atorvastatin protected the rat brain against focal ischemia through the downregulation of HMGB1, TLR4 and NF-κB expression [34]. HMGB1 was also shown to be involved in traumatic brain injury (TBI) [35]. Anti-HMGB1 mAb inhibited the expression of inflammatory molecules and brain edema through the protection of blood-brain barrier (BBB) function and improved motor function after TBI [35]. After spinal cord injury, HMGB1 was shown to be produced by both activated macrophages and neurons at early phases [36]. The inhibition of HMGB1 reduced cell swelling in spinal cord astrocytes after oxygen and glucose deprivation and reoxygenation through HMGB1/TLR4/MyD88/NF-κB signalling [37]. Our data confirmed that a correlation exist between the concentrations of HMGB1 and IL-6 in CSDH fluids. Considering all together, HMGB1 might regulate the growth of CSDH outer membranes through the TLR4/NF-κB signaling pathway (Fig 4). This HMGB1 might be a therapeutically attractive target for the treatment of intractable CSDH.

This study has several limitations. First, our findings were purely observational. We explored the expression of TLR and NF-κB signaling molecules in CSDH outer membranes. Additional definitive experiments are required to determine whether these molecules are activated during the growth of CSDH outer membranes. Second, no control cases, such as patients with chronic subdural effusion, were included to compare the expression levels of these molecules. Further studies are necessary to explore the complicated relationship between TLR4, NF-κB and the MAPK signaling pathway, which have previously been implicated in the growth of CSDH outer membranes [38, 39].

## Conclusions

We have identified the expression of TLR signaling pathway molecules in CSDH outer membranes for the first time. TLR4, NF-κB/p65 and IL-6 were expressed in endothelial cells. The TLR signaling pathway may be activated by HMGB1 and produce IL-6 through the NF-κB signaling pathway, which precisely regulates the growth of CSDH outer membranes, although additional molecules may also be involved in the growth of CSDH. Further clinical trials targeting members of this TLR signaling pathway, especially HMGB1, are needed to help develop treatments for recurrent and intractable CSDH.

## Supporting information

S1 Raw Images Raw data of Fig 1. (ZIP)
