# Supplementary figures and images for: Expression of high mobility group B1 and toll-like receptor-nuclear factor κB signaling pathway in chronic subdural hematomas
Source: PLoS One. 2020 Jun 1;15(6):e0233643. doi: 10.1371/journal.pone.0233643 (PMC7263617; doi:10.1371/journal.pone.0233643)

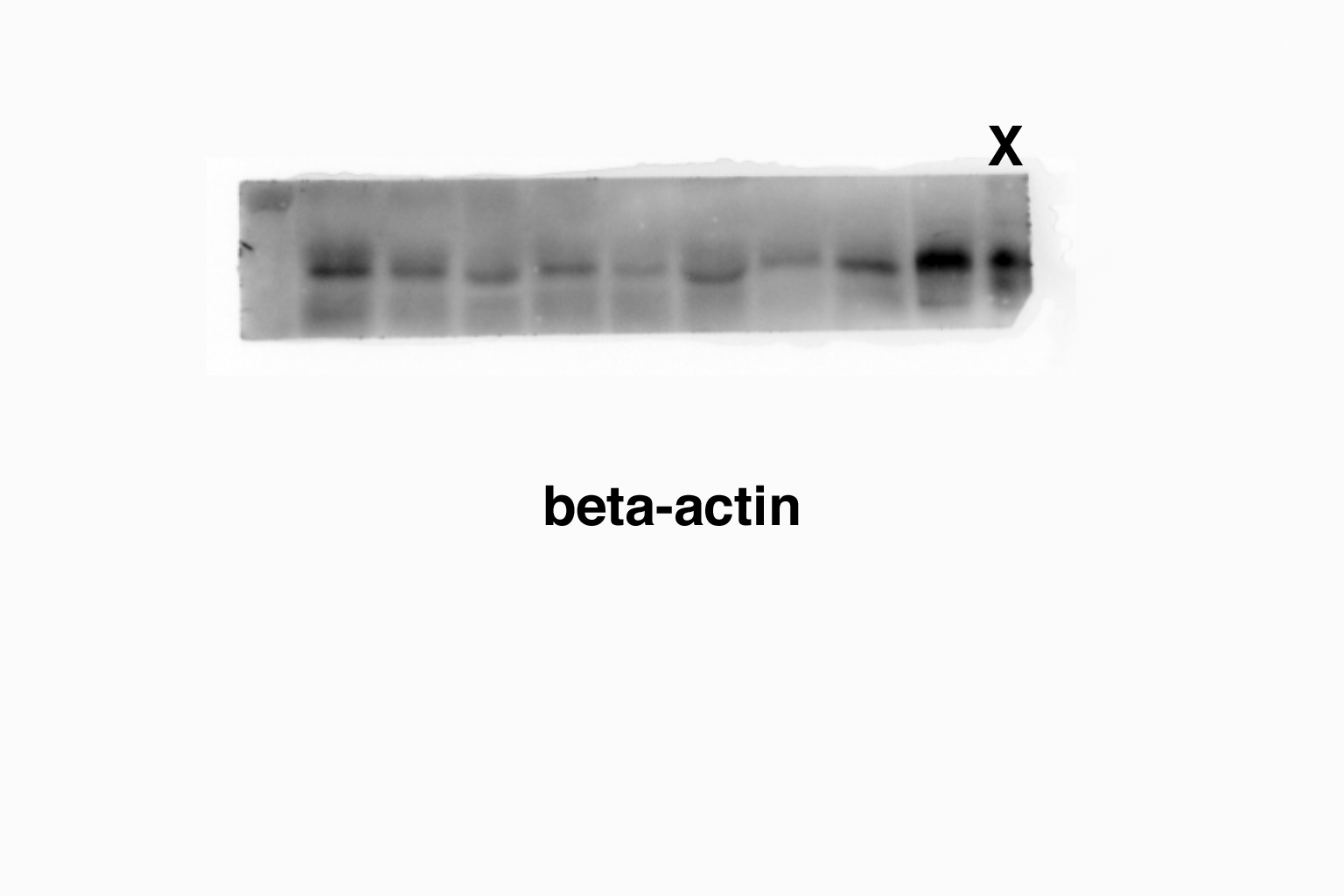

Supplement: S1 Raw Images — (ZIP) [file pone.0233643.s001.zip › S1 raw image/beta-actin.tif]

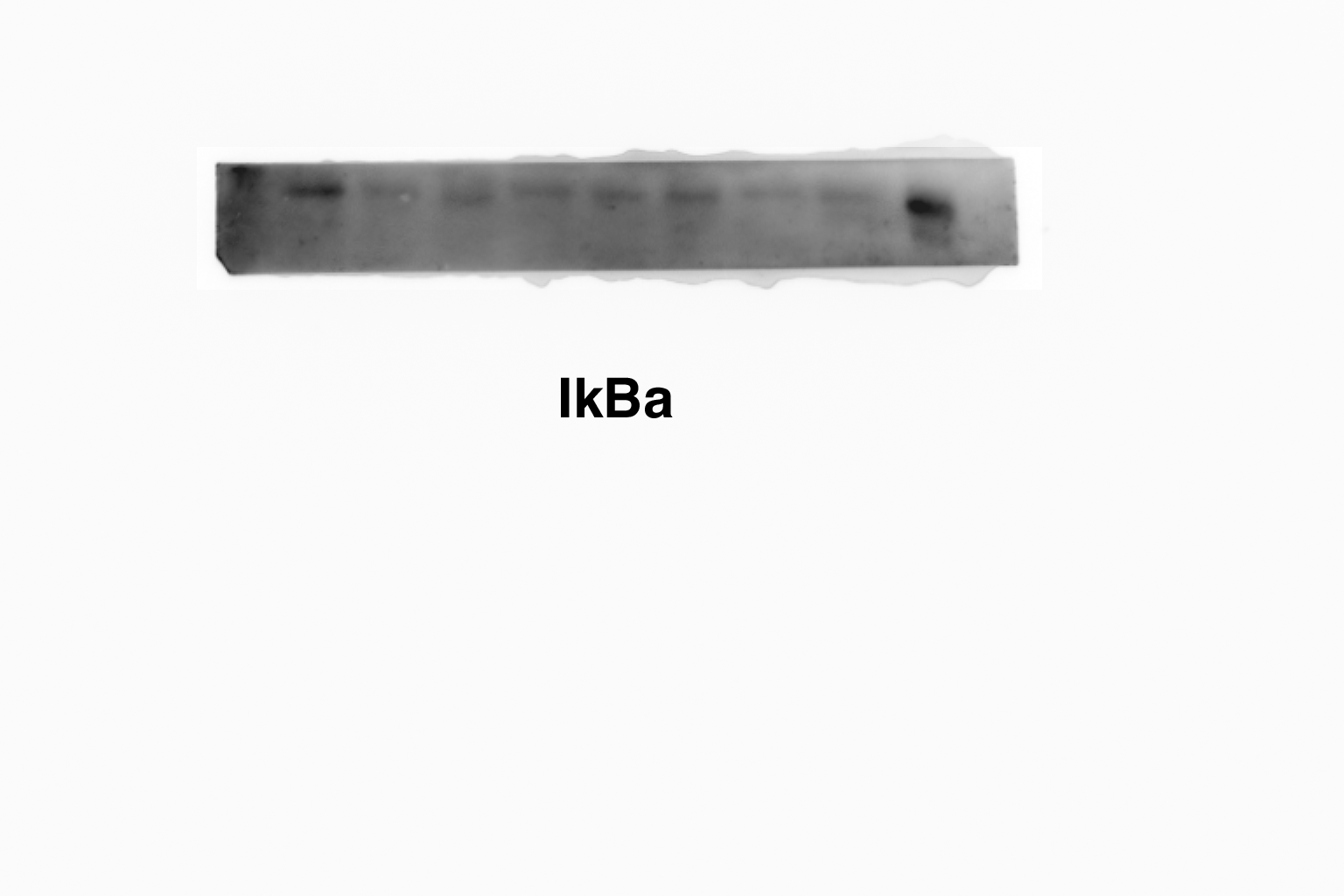

Supplement: S1 Raw Images — (ZIP) [file pone.0233643.s001.zip › S1 raw image/IkBa.tif]

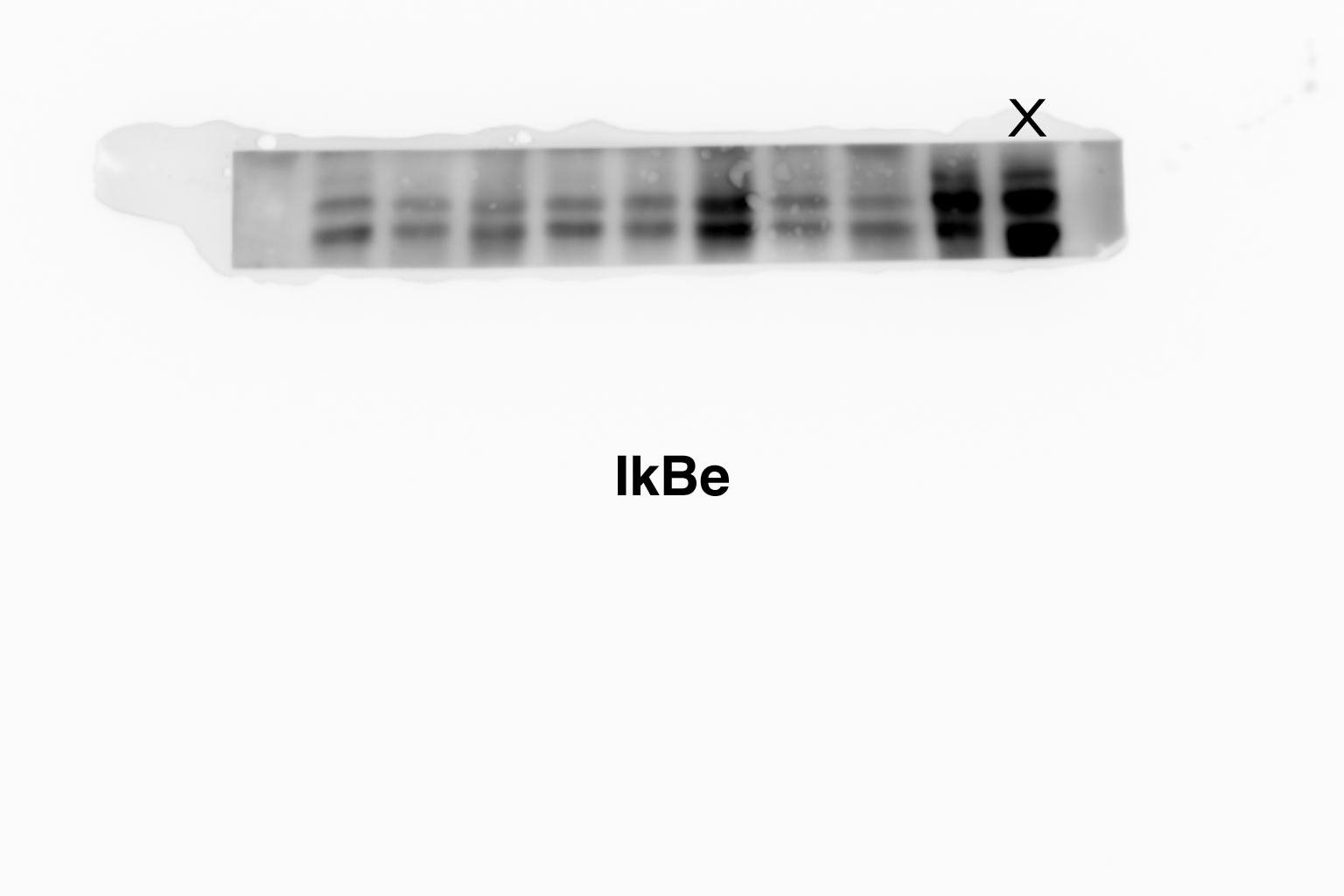

Supplement: S1 Raw Images — (ZIP) [file pone.0233643.s001.zip › S1 raw image/IkBe.tif]

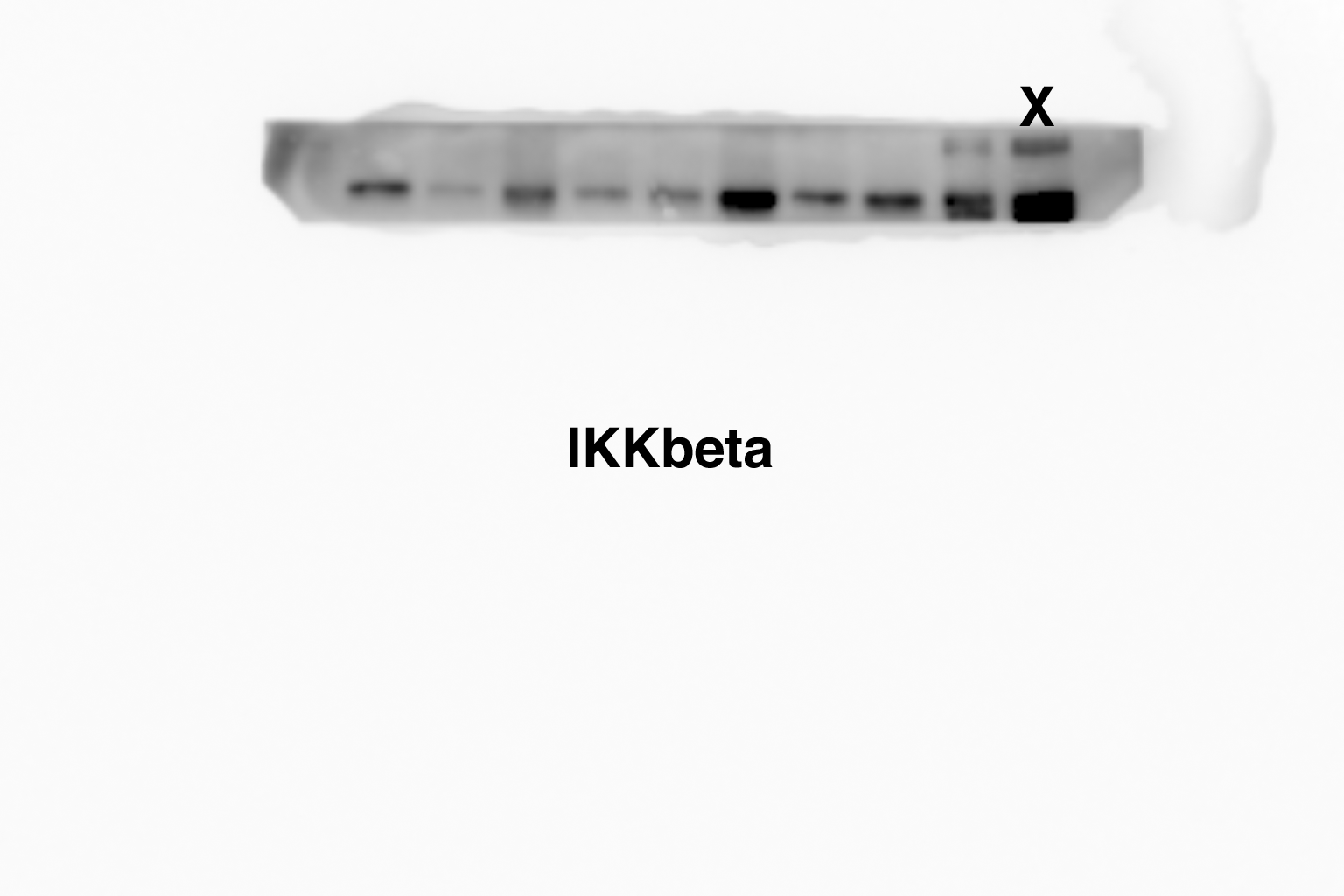

Supplement: S1 Raw Images — (ZIP) [file pone.0233643.s001.zip › S1 raw image/IKKbeta.tif]

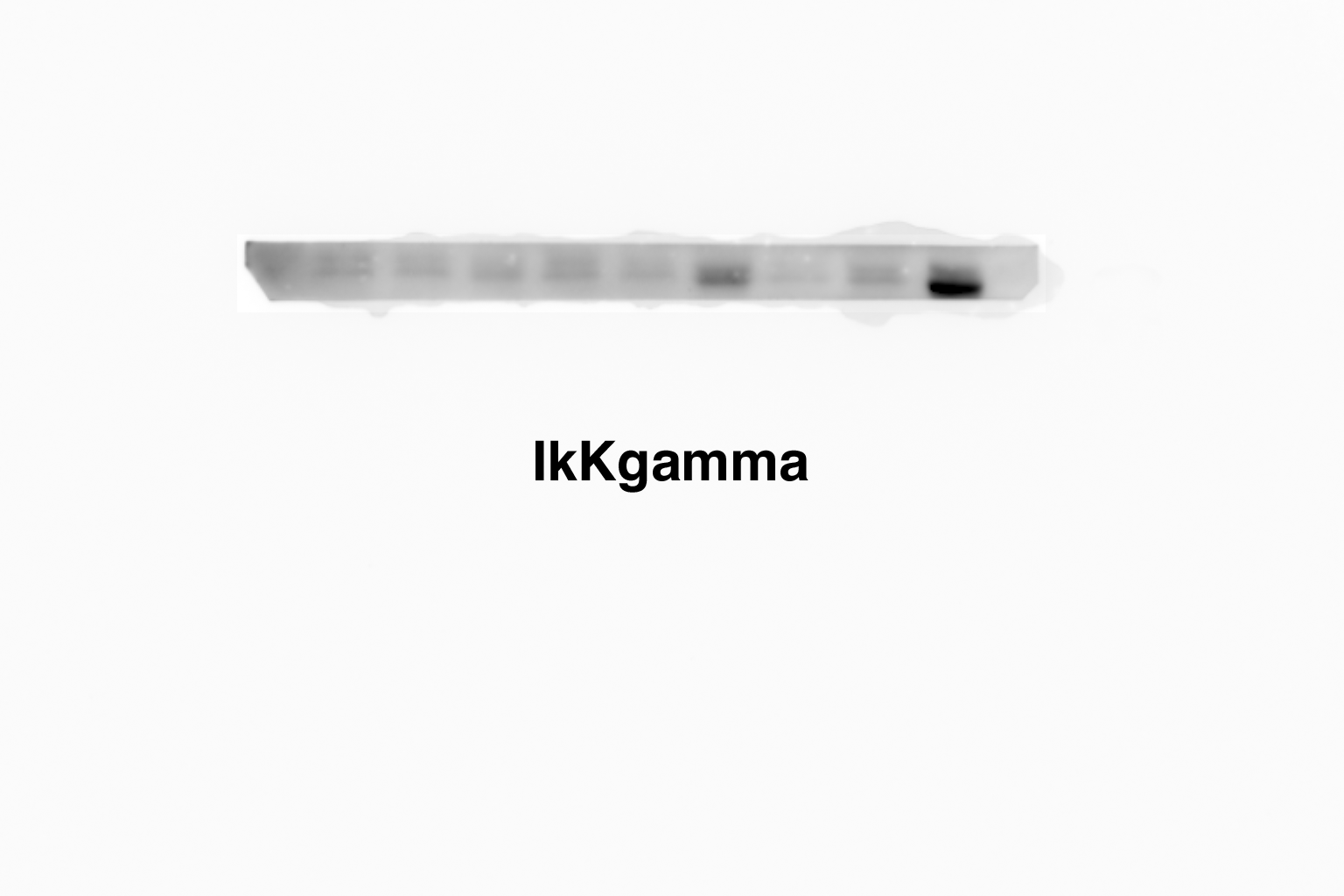

Supplement: S1 Raw Images — (ZIP) [file pone.0233643.s001.zip › S1 raw image/Ikkgamma.tif]

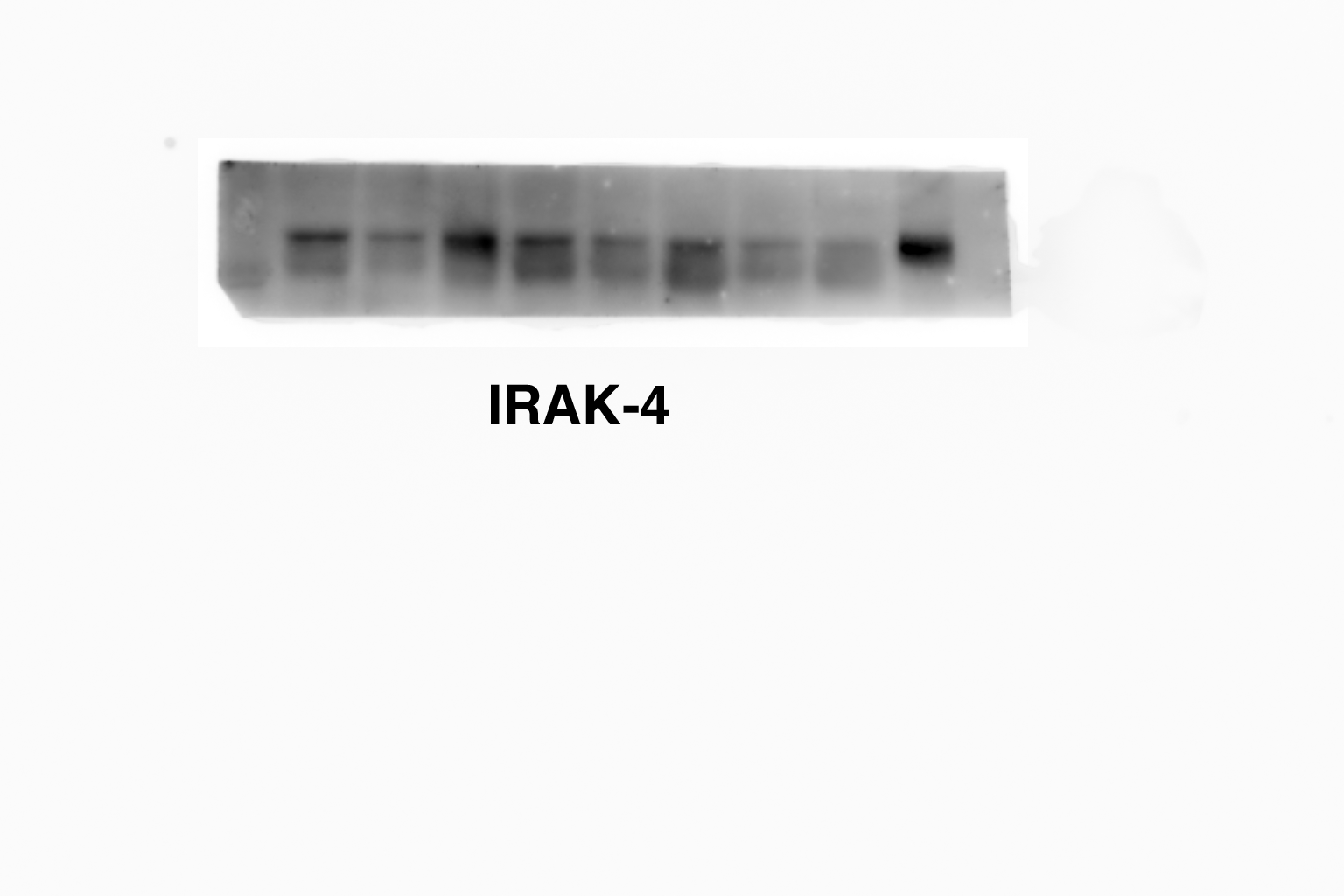

Supplement: S1 Raw Images — (ZIP) [file pone.0233643.s001.zip › S1 raw image/IRAK4.tif]

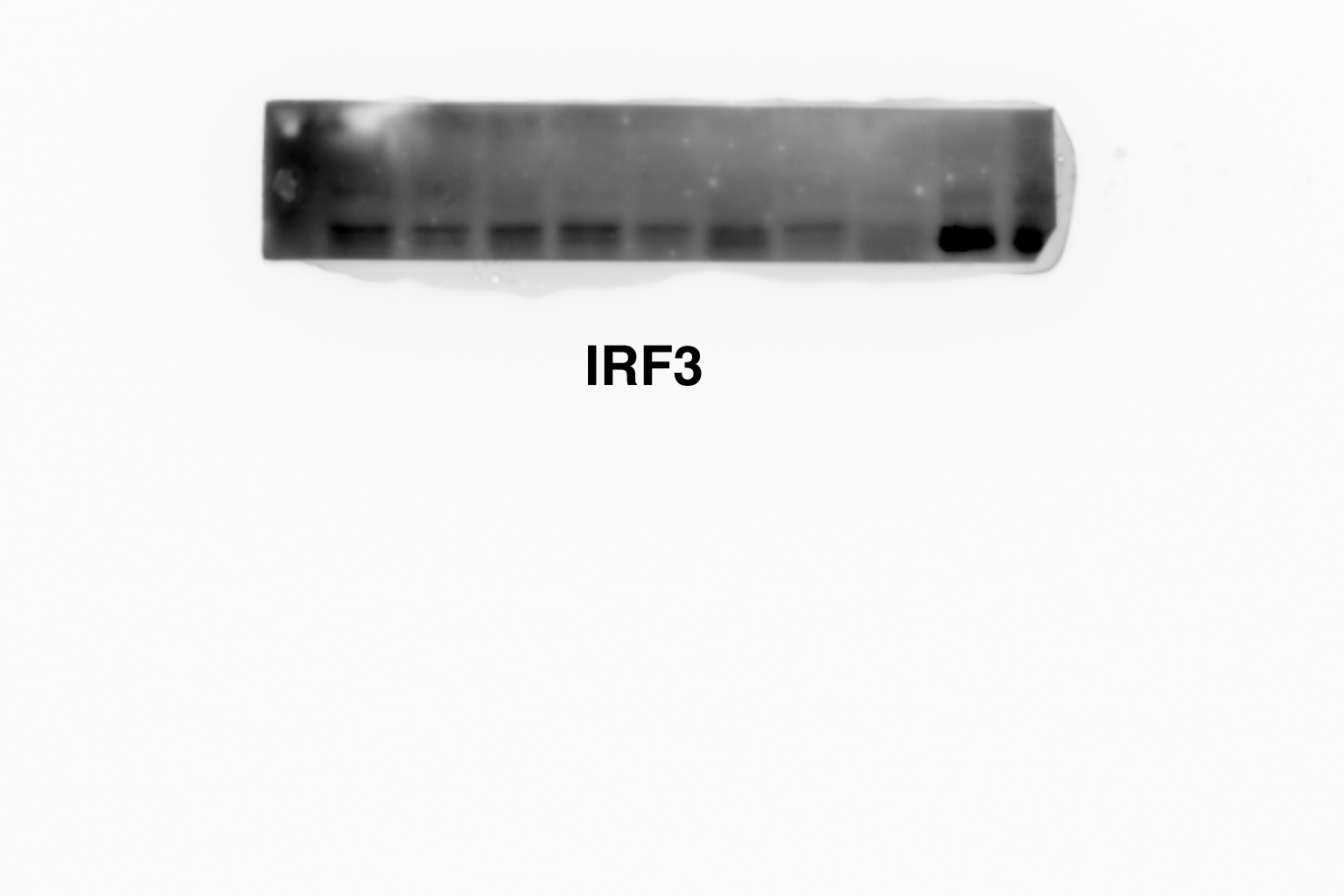

Supplement: S1 Raw Images — (ZIP) [file pone.0233643.s001.zip › S1 raw image/IRF3.tif]

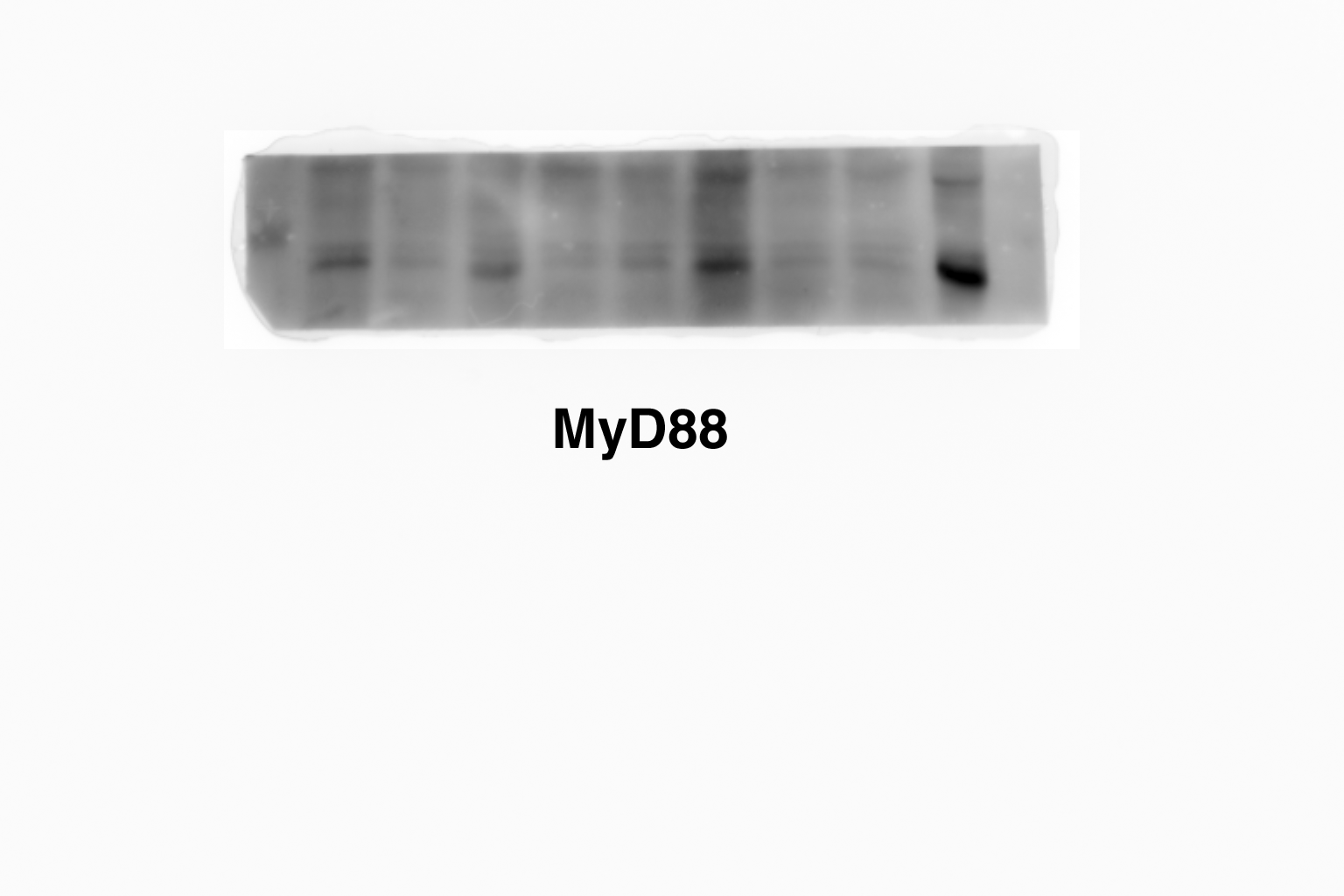

Supplement: S1 Raw Images — (ZIP) [file pone.0233643.s001.zip › S1 raw image/MyD88.tif]

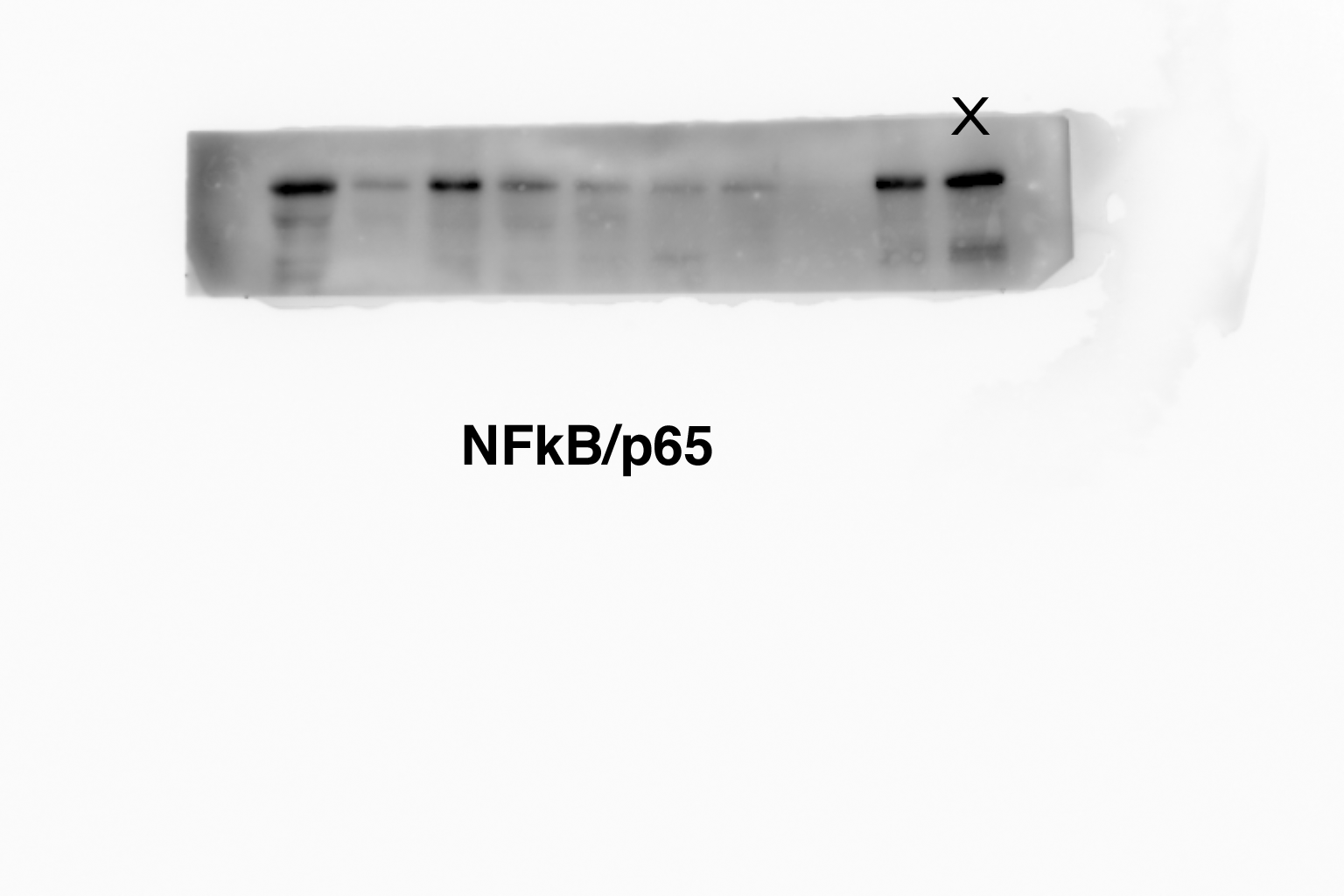

Supplement: S1 Raw Images — (ZIP) [file pone.0233643.s001.zip › S1 raw image/NFkBp65.tif]

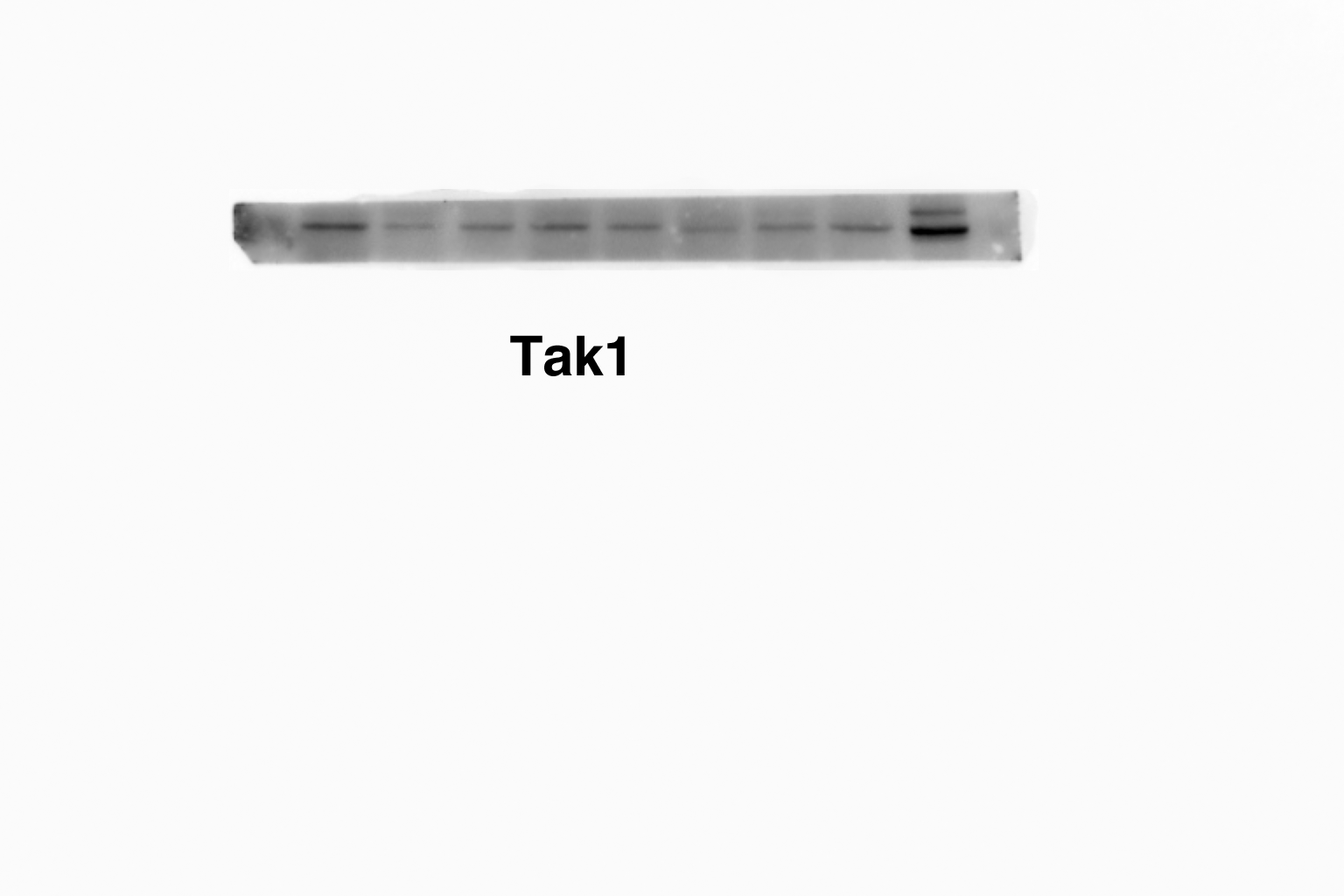

Supplement: S1 Raw Images — (ZIP) [file pone.0233643.s001.zip › S1 raw image/Tak1.tif]

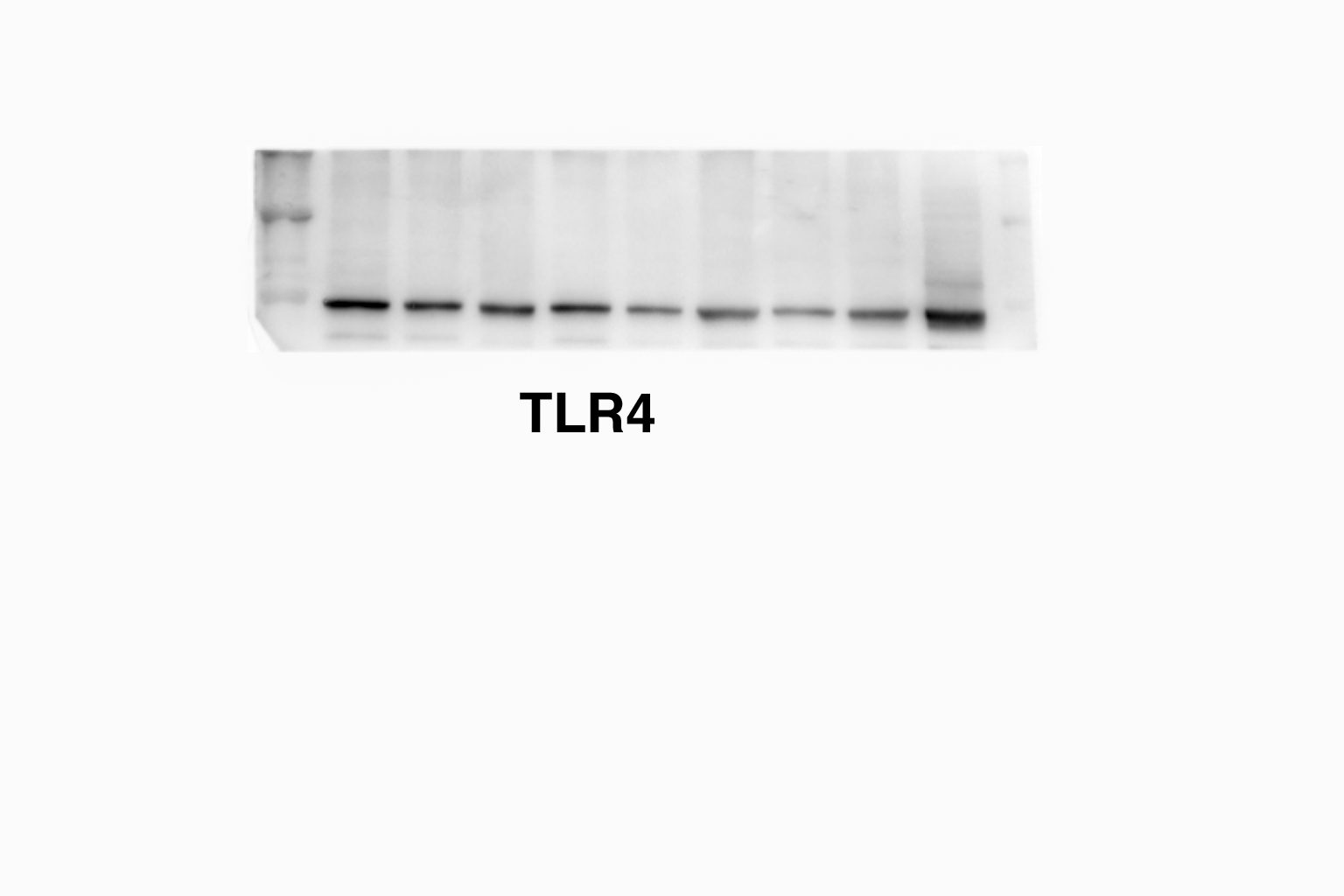

Supplement: S1 Raw Images — (ZIP) [file pone.0233643.s001.zip › S1 raw image/TLR4.tif]

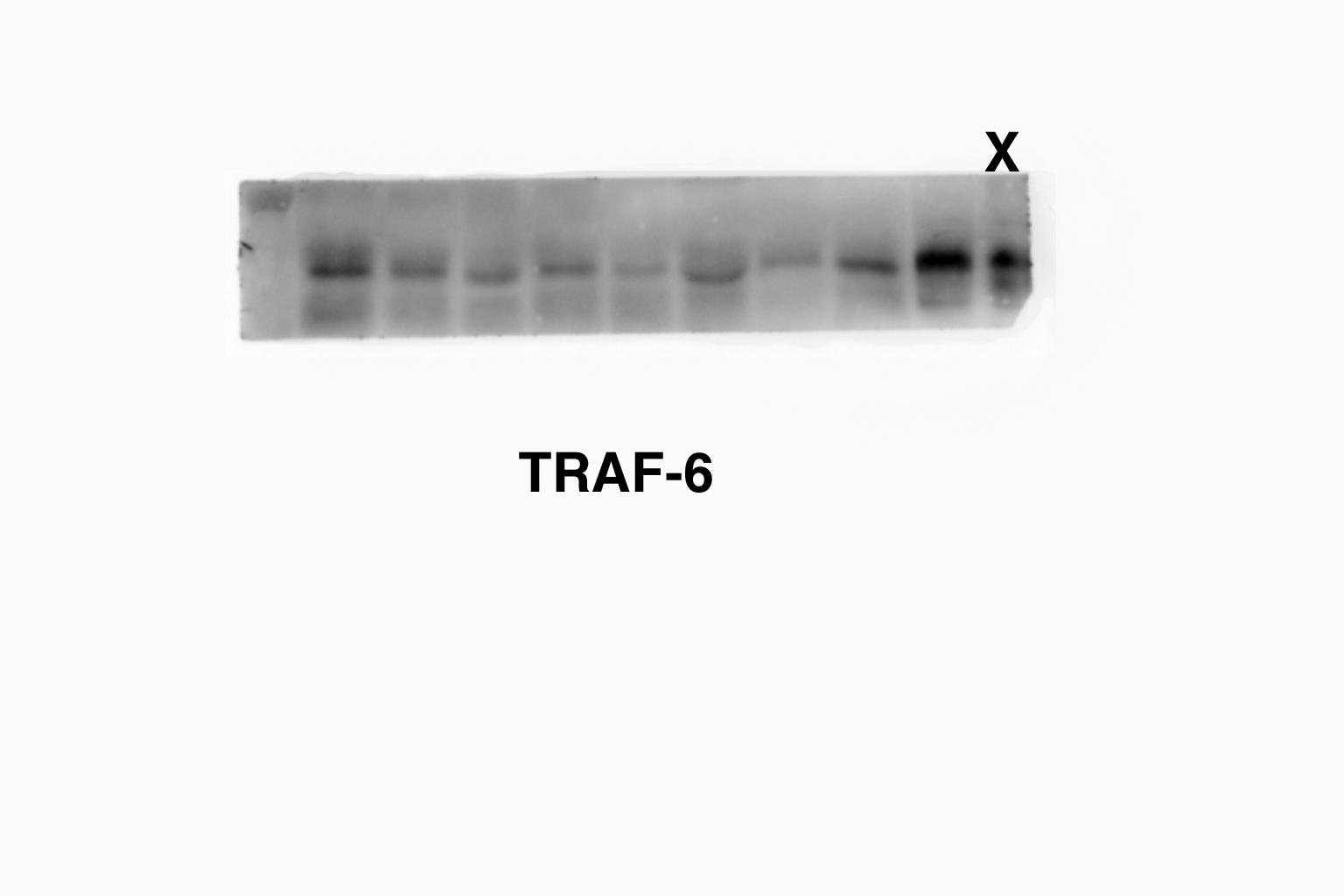

Supplement: S1 Raw Images — (ZIP) [file pone.0233643.s001.zip › S1 raw image/TRAF-6.tif]
